# Depletion of senescent-like neuronal cells alleviates cisplatin-induced peripheral neuropathy in mice

**DOI:** 10.1038/s41598-020-71042-6

**Published:** 2020-08-25

**Authors:** Scarlett Acklin, Manchao Zhang, Wuying Du, Xin Zhao, Matthew Plotkin, Jianhui Chang, Judith Campisi, Daohong Zhou, Fen Xia

**Affiliations:** 1 Department of Radiation Oncology, University of Arkansas for Medical Sciences, Little Rock, AR 72205 USA; 2 Division of Nephrology, University of Arkansas for Medical Sciences, Little Rock, AR 72205 USA; 3 Department of Pharmaceutical Sciences, University of Arkansas for Medical Sciences, Little Rock, AR 72205 USA; 4 Buck Institute for Research on Aging, Novato, CA USA; 5 Lawrence Berkeley National Laboratory, Berkeley, CA USA; 6 Department of Pharmacodynamics and Department of Radiation Oncology, University of Florida, Gainesville, FL 32611 USA

**Keywords:** Senescence, Neurotoxicity syndromes, Chemotherapy

## Abstract

Chemotherapy-induced peripheral neuropathy is among the most common dose-limiting adverse effects of cancer treatment, leading to dose reduction and discontinuation of life-saving chemotherapy and a permanently impaired quality of life for patients. Currently, no effective treatment or prevention is available. Senescence induced during cancer treatment has been shown to promote the adverse effects. Here, we show that cisplatin induces senescent-like neuronal cells in primary culture and in mouse dorsal root ganglia (DRG), as determined by the characteristic senescence markers including senescence-associated beta-galactosidase, accumulation of cytosolic p16^INK4A^ and HMGB1, as well as increased expression of p16Ink4a, p21, and MMP-9. The accumulation of senescent-like neuronal cells in DRG is associated with cisplatin-induced peripheral neuropathy (CIPN) in mice. To determine if depletion of senescent-like neuronal cells may effectively mitigate CIPN, we used a pharmacological ‘senolytic’ agent, ABT263, which inhibits the anti-apoptotic proteins BCL-2 and BCL-xL and selectively kills senescent cells. Our results demonstrated that clearance of DRG senescent neuronal cells reverses CIPN, suggesting that senescent-like neurons play a role in CIPN pathogenesis. This finding was further validated using transgenic p16-3MR mice, which permit ganciclovir (GCV) to selectively kill senescent cells expressing herpes simplex virus 1 thymidine kinase (HSV-TK). We showed that CIPN was alleviated upon GCV administration to p16-3MR mice. Together, the results suggest that clearance of senescent DRG neuronal cells following platinum-based cancer treatment might be an effective therapy for the debilitating side effect of CIPN.

## Introduction

Chemotherapy-induced peripheral neuropathy, a common dose-limiting toxicity of many chemotherapy regimens, limits the potentially curative effects of systemic chemotherapy^1–4^. Particularly, platinum-based chemotherapeutics, such as cisplatin, are known to cause systemic neuronal toxicity. Clinically, cisplatin-induced peripheral neuropathy (CIPN) presents as burning, shooting or electric-shock-like pain affecting the feet and hands^2,5^, for which no effective treatments or preventive measures are available^2–4,6^.

Cisplatin’s antineoplastic effects are achieved through the formation of platination products in nuclear DNA^7–9^. These DNA-base crosslinks distort DNA helices, interrupting replication and transcription, eventually inducing cell cycle arrest, senescence, or cell death^7–10^.While these effects of cisplatin on cancer cells is desired for treatment, the same processes in normal tissue can cause toxicity.

An important senescence phenotype, termed therapy-induced senescence (TIS), can be induced by DNA damage-based chemotherapeutics. The genotoxic stress caused by these agents induces senescence during cancer treatment and has been shown to promote the adverse effects of chemotherapy^11,12^. Cellular senescence, a conserved response to stress, results in a stable cell cycle arrest while maintaining cell viability and metabolic activity^13,14^. The distinct metabolic and signaling features of senescent cells include a senescence-associated secretory phenotype (SASP). The expression of SASP includes the secretion of numerous molecules, including growth factors, proteases, cytokines, chemokines and extracellular matrix components, which mediate the paracrine activities of senescent cells^13,15–17^. Despite the relatively low proportion of senescent cells in tissues, the SASP allows these cells to generate durable local and systemic deleterious effects in most tissues, which contribute to the pathogenesis of a variety of diseases including chemotherapy toxicity^11^.

We hypothesized that senescence and the SASP might also play a role in CIPN following neuronal DNA damage, and the depletion of senescent cells may be an effective treatment of peripheral neuropathy induced by cisplatin. We showed that cisplatin induces peripheral neuropathy, as confirmed by mechanical and thermal pain assessment, and was associated with the accumulation of senescent-like neuronal cells in the dorsal root ganglia (DRG) using immunostaining and qPCR for senescence biomarkers. Furthermore, we provided genetic and pharmacologic evidence that selective clearance of senescent-like DRG neurons alleviates CIPN. Together, our results suggest that senolytic agents have the potential to be a novel therapeutic strategy for CIPN.

## Results

### Cisplatin induces senescence-like response in DRG neurons

Hypersensitivity to mechanical and thermal stimuli is well-documented following damage to DRG sensory neurons^18–20^. In CIPN, DRG could be particularly important in part due to a lack of the blood–brain barrier in the peripheral nervous system and resulting exposure to endogenous and exogenous agents. In fact, prior studies showed preferential binding of cisplatin to DNA in DRG with a high platinum adduct formation^9,21,22^.

**Figure 1:**
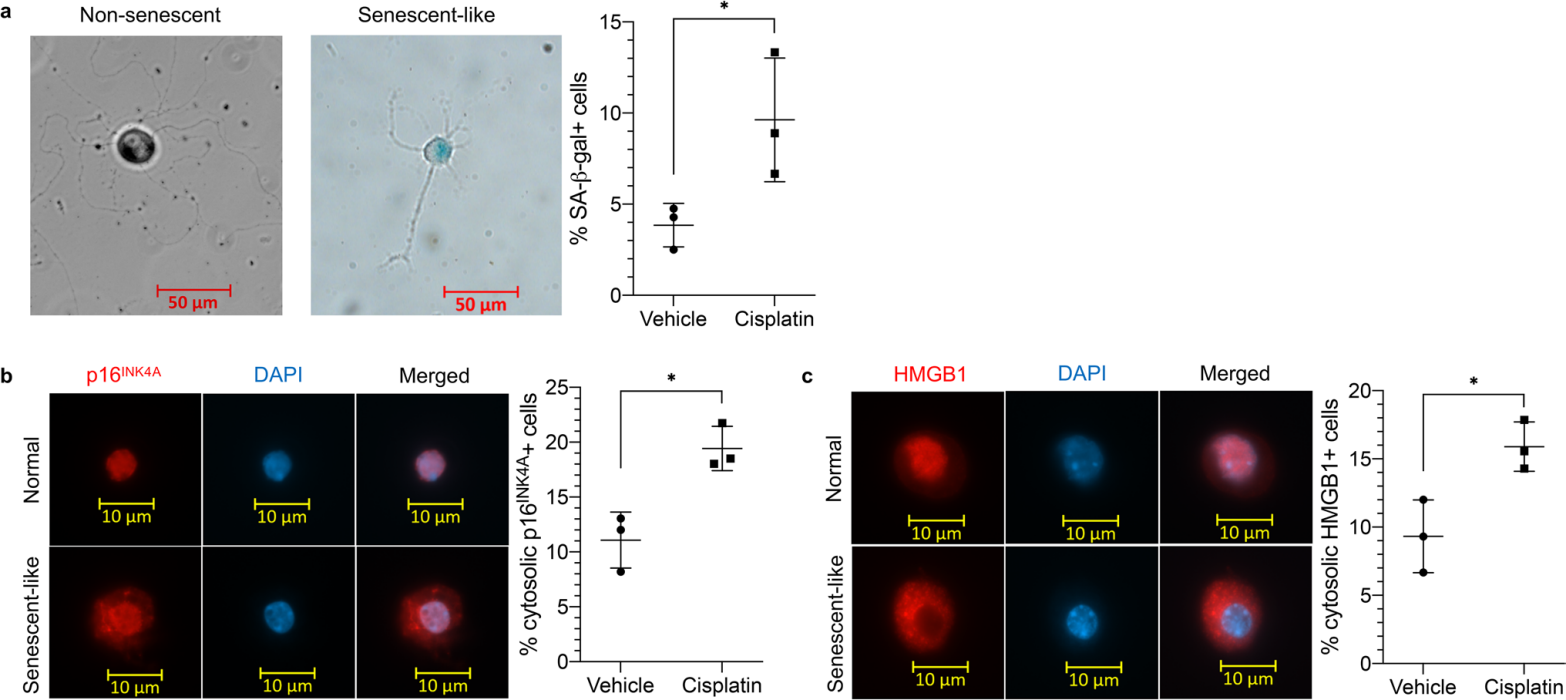
Cisplatin induces a senescence-like response in DRG neurons. (**a**) Representative images of SA-β-gal stained primary DRG neurons cultured from WT C57BL/6 mice. Percent SA-β-gal positive cells was calculated as the number of neurons with positive SA-β-gal staining divided by the total number of cells left on the coverslip times 100 (n = 3). *P* = 0.0497. (**b**) p16^INK4A^ was detected by anti-p16^INK4A^ immunofluorescence (red). Percent cytosolic p16^INK4A^ positive cells was calculated as the number of neurons with cytosolic p16^INK4A^ immunofluorescence, visualized by DAPI nuclei staining (blue), divided by the total number of cells left on the coverslip times 100 (n = 3). *P* = 0.0113. (**c**) Visualization of HMGB1 as detected by anti-HMGB1 immunofluorescence (red). Percent cytosolic HMGB1 positive cells was calculated as the number of neurons with cytosolic HMGB1 immunofluorescence divided by the total number of cells left on the coverslip times 100 (n = 3). Data points are mean values ± SEM and were analyzed by two-tailed Student’s t-test.

Although neurons are constitutively post-mitotic, there is increasing evidence they can undergo a senescence-like response^23–26^. To determine whether senescent cells play a role in CIPN, we first investigated whether cisplatin induces a senescence-like state in DRG by localizing and measuring biomarkers for senescent cells in cultured primary DRG neurons following cisplatin treatment. Because DRG are affected in peripheral neuropathy and are vulnerable to the cytotoxic effects of systemic drugs, localization of senescent cells here is particularly relevant. We treated primary cultured neurons isolated from adult C57BL/6 mice with the clinically relevant dose of cisplatin used in the CIPN model and analyzed the cells for senescence-associated β-galactosidase (SA-β-gal) staining, a widely used biomarker for senescent cells^23,27^. Following cisplatin exposure, significantly more neurons stained positive for SA-β-gal compared to vehicle-treated cells (Fig. 1a, *P* = 0.0497), suggesting that cisplatin induces senescence in DRG.

p16^INK4A^, a cyclin-dependent kinase inhibitor also frequently used as a senescence-associated marker^28–30^, has been shown to accumulate in the cytoplasm of cells following doxorubicin-induced senescence^31^. Immunofluorescence staining for p16^INK4A^ in primary DRG neurons also showed cytoplasmic accumulation following treatment with cisplatin for 5 days . Compared to vehicle-treated neurons, those treated with cisplatin exhibited significantly greater cytoplasmic p16^INK4A^ (Fig. 1b, *P* = 0.0113).

Similarly, senescent human and mouse cells have been shown to relocalize nuclear HMGB1 (high mobility group box 1) in a p53-dependent manner^32^. Primary DRG neurons were treated with the same clinically relevant dose of cisplatin for 5 days and stained for HMGB1. Cisplatin-treated cells showed significantly increased HMGB1 in the cytosol compared to vehicle-treated cells (Fig. 1c, *P* = 0.0241).

### Cisplatin-induced senescence-like response in DRG neurons is associated with CIPN in murine model

**Figure 2:**
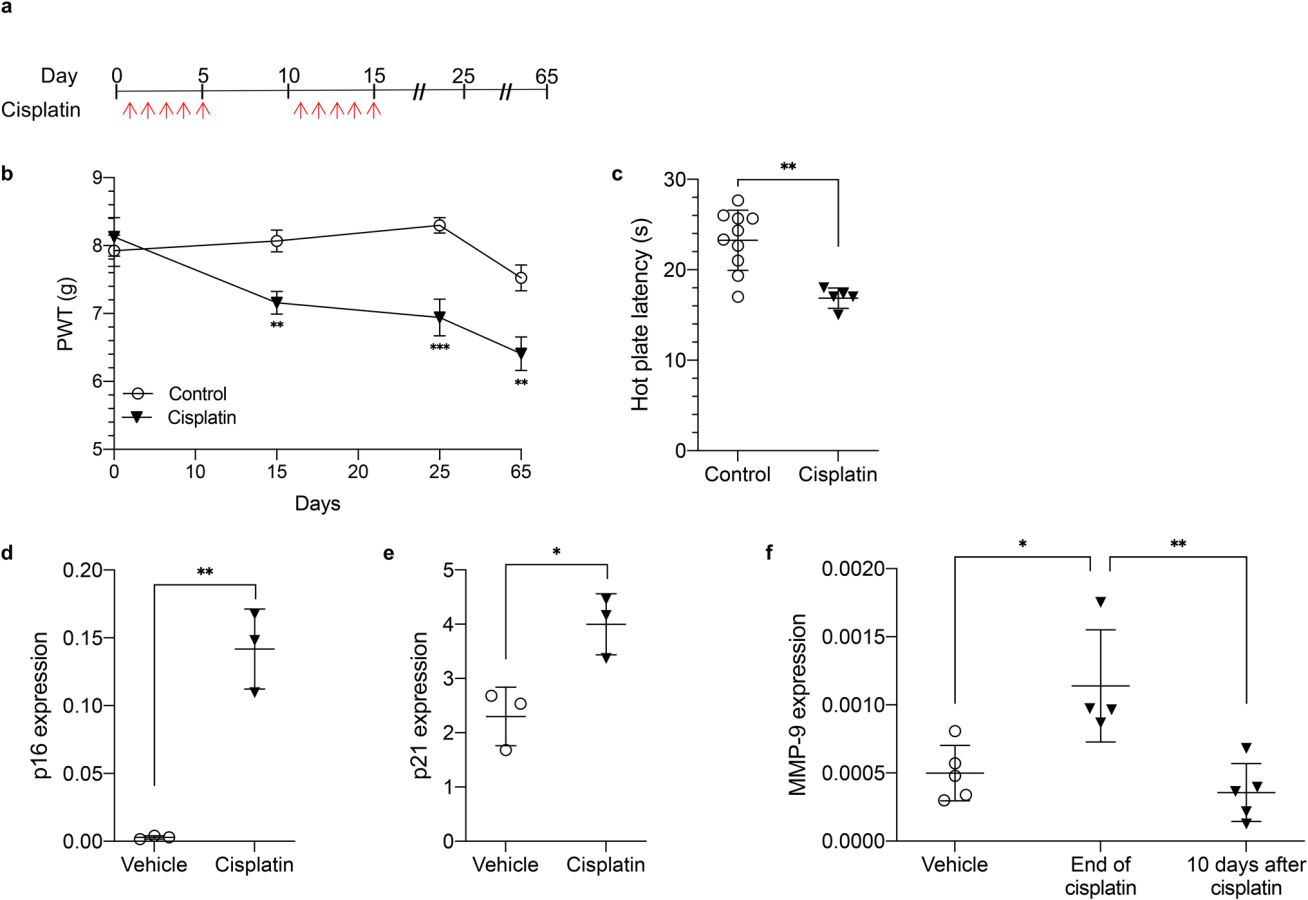
Cisplatin-induced senescence-like response in DRG neurons is associated with CIPN in murine model. (**a**) Cisplatin regimen for inducing peripheral neuropathy. (**b**) Mechanical allodynia measured in wild type (WT) mice by von Frey tests after treatment with 2.3 mg/kg cisplatin (*P* = 0.0039, 0.0001, and 0.0040). (**C**) Thermal algesia measured by hot plate tests in WT mice 51 days after cisplatin treatment (*P* = 0.0012). In the control and cisplatin groups, n = 10 and n = 5, respectively. (**d**) p16Ink4a (*P* = 0.0012) and (**e**) p21 expression (*P* = 0.0196) in C57BL/6 mice DRG neurons 17 days after cisplatin treatment (n = 3). (**a–e**) Data points are mean values ± SEM and were analyzed by two-tailed Student’s t-test. (**f**) Quantification of *Mmp-9* mRNA levels following saline treatment or 0 and 10 days after cisplatin treatment (n = 5). *P* = 0.015 and 0.004. Data points are mean values ± SEM and were analyzed by one-way ANOVA with post-hoc Tukey test.

To determine if the induced senescence-like response contributes to CIPN, we first established and characterized a CIPN mouse model in adult C57BL/6 mice. To mimic a clinical treatment schedule, we gave mice a clinically relevant dose of cisplatin (2.3 mg/kg^33–35^) or saline by a daily intraperitoneal (*i.p.*) injection for 5 days, followed by 5 days of rest, and then the second 5-day cycle of cisplatin treatment (Fig. 2a). Because common manifestations of CIPN in patients include hyperalgesia^3,6,10^, we assessed mechanical thresholds (i.e. paw-withdrawal pressure in grams) with von Frey filaments and thermal thresholds (i.e., paw-withdrawal latency in seconds) using dynamic hot plates to monitor the development of CIPN before and at varying intervals after cisplatin treatment. The mechanical thresholds were tested on days 0, 15, 25 and 65, and the thermal thresholds were measured at a single time point on day 66. The results showed that control mice receiving saline retained relatively stable mechanical allodynia thresholds during and following the treatment course, showing no substantial difference in mechanical thresholds compared to baseline values. Consistent with CIPN, mice receiving cisplatin displayed significant decreases in mechanical threshold at days 15, 25, and 65 (Fig. 2b; *P* = 0.003880, 0.000107, and 0.003987 respectively). They also exhibited significantly higher sensitivity to mechanical stimuli compared to baseline measurements as well as to control mice. Likewise, cisplatin-treated mice demonstrated decreased hot plate latency on day 66 compared to control mice, a finding consistent with thermal hyperalgesia (Fig. 2c, *P* = 0.0012). There was no substantial difference between females and males observed in mechanical or thermal sensitivity (data not shown). Together, these results demonstrate that cisplatin-treated mice developed CIPN as measured by mechanical and thermal allodynia.

To verify that these findings were associated with development of a senescence-like state, we measured known senescence markers in the DRG of the mice. A well-established hallmark of senescent cells is increased expression of p16Ink4a^36^, encoded by the *Cdkn2a* locus, often resulting from persistent DNA damage and activation of the p38 pathway^37^. We measured *Cdkn2a* mRNA levels in DRG 17 days after the end of the second cisplatin cycle. When compared to mice treated with vehicle, cisplatin-treated mice showed a significant increase in *Cdkn2a* mRNA levels in their DRG (Fig. 2d, *P* = 0.0012). Increased expression of another classic senescence marker, p21Cip1/WAF^38^, has been indicated as the crucial signal transducer between the response to DNA damage and senescence-like changes in postmitotic neurons^25^. Measurement of p21 expression in mice DRG 17 days after cisplatin treatment revealed a significant increase in gene expression compared to vehicle-treated mice (Fig. 2e, *P* = 0.0196).

Senescent cells express numerous SASP factors, including proteases, cytokines, chemokines and matrix metalloproteinases (MMPs,^12,14,39–41^), which could mediate CIPN. Therefore, we measured SASP factors in DRG from mice treated with cisplatin. While many known SASP factors showed no considerable change (not shown), which can occur when senescence is induced primarily by p16Ink4a^38^, *Mmp-*9 mRNA levels increased after cisplatin treatment and returned to basal levels 10 days later (Fig. 2f, *P* = 0.015 and 0.004). These findings along with SA-β-gal, cytosolic p16^INK4A^, and cytosolic HMGB1 staining and p16Ink4a expression indicate that cisplatin induces a senescent-like state in primary DRG neurons.

### Genetic clearance of senescent cells reverses CIPN in mice

**Figure 3:**
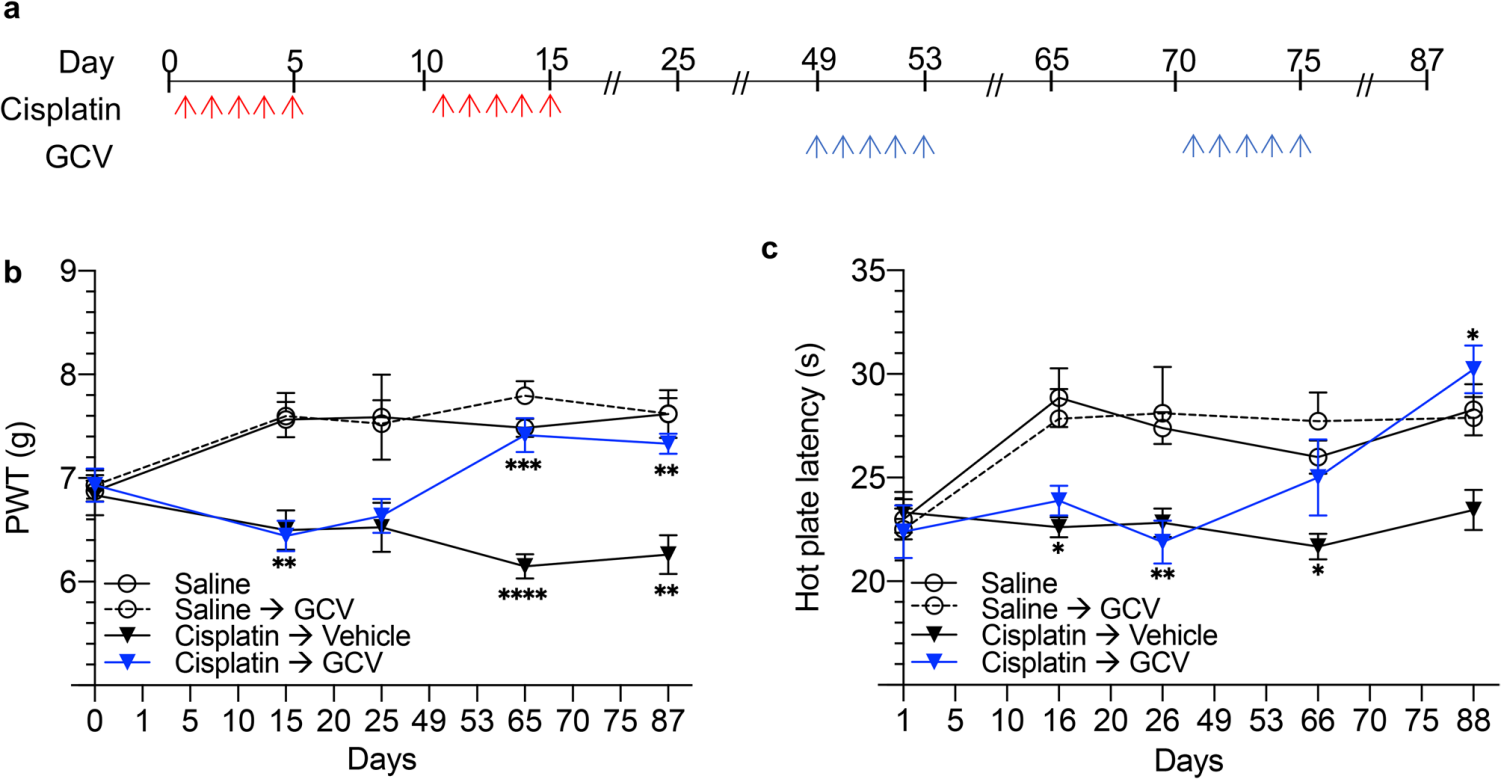
Genetic clearance of senescent cells reverses CIPN in mice. (**a**) Cisplatin and GCV treatment regimen to induce CIPN and eliminate senescent cells, respectively, in p16-3MR mice. (**b**, **c**) Response of p16-3MR mice, which are sensitive to GCV-mediated senescent cell clearance, to (**b**) mechanical and (**c**) thermal stimulation before and after cisplatin treatment. Cisplatin-treated mice also underwent senescent cell clearance via GCV treatment. Mechanical sensitivity of cisplatin-treated mice was compared to saline-treated (*P* = 0.0091, *P* < 0.0001, and *P* = 0.0077) and cisplatin-treated mice following senescent cell removal (*P* = 0.0007 and 0.0052). Thermal sensitivity of cisplatin-treated mice was also compared to saline-treated (*P* = 0.0323, 0.0084, and 0.0128) and cisplatin-treated mice treated with GCV (*P* = 0.0255). Data points are mean values ± SEM and were analyzed by two-way ANOVA analysis with post-hoc Tukey test. n = 6.

To confirm the role of senescent neuronal cells in CIPN, we next examined whether removal of senescent cells reverses CIPN by taking advantage of the p16-3MR transgene in genetically modified mice. Senescent cells in p16-3MR mice express the herpes simplex virus 1 thymidine kinase (HSV-TK), thereby sensitizing p16Ink4a-expressing cells to ganciclovir (GCV,^12,40^). We induced CIPN in p16-3MR mice as described in Fig. 2, and control mice received saline injections. Following CIPN development, cisplatin-treated mice received daily *i.p.* injections of GCV on days 49–53 and again on days 70–74 (Fig. 3a). Mechanical thresholds were measured on days 0, 15, 25, 65, and 87 following cisplatin and after each cycle of GCV. Thermal thresholds were measured 1 day after each mechanical threshold measurement. GCV, in the absence of cisplatin, had no effect on mechanical (Fig. 3b) or thermal (Fig. 3c) thresholds compared to control p16-3MR mice receiving only saline. Interestingly, administration of GCV to cisplatin-treated mice reversed CIPN as assessed by a restoration of mechanical threshold on day 65 and day 87 (Fig. 3b). Cisplatin similarly decreased thermal thresholds, which were restored by GCV treatment (Fig. 3c). No considerable difference between females and males was observed in mechanical or thermal sensitivity (data not shown). Together, these data provide genetic evidence that senescent cells contribute to CIPN, and depletion of senescent cells reverses CIPN.

### Pharmacological clearance of senescent cells with ABT263 reverses CIPN in mice

Previous studies showed that induction of senescence by chemotherapy contributes to cancer metastasis and recurrence and treatment-related toxicity such as bone marrow suppression and cardiac dysfunction^11,13,14,39^. Senescence-targeted therapy using one of three approaches—senolysis to permanently remove senescent cells, inhibition of SASP expression with a senomorphic therapeutic, and promotion of immune-mediated senescent cell clearance^14,39^—has the potential to prevent, mitigate and perhaps reverse chemotherapy-induced toxicity including CIPN.

**Figure 4:**
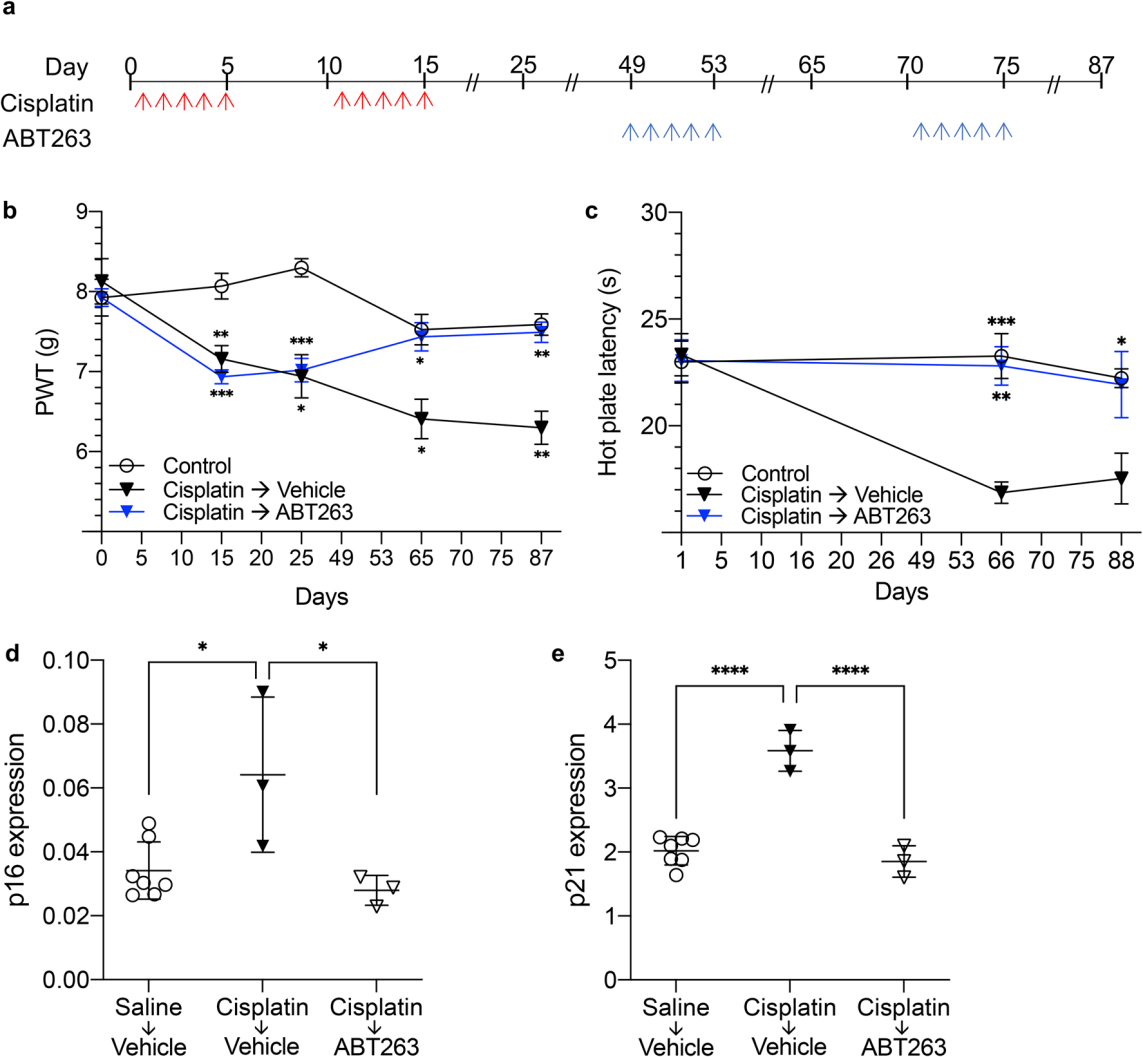
Pharmacological clearance of senescent cells with ABT263 reverses CIPN in mice. (**a**) Treatment regimen to induce CIPN and then remove senescent cells with ABT263 in C57BL/6 mice. (**b**) Response of WT mice to mechanical stimulation, as measured by von Frey tests, before and after treatment with 2.3 mg/kg cisplatin (n = 5, *P* = 0.0067, 0.0103, 0.0153, and 0.0027 on days 15, 25, 65, and 87 for cisplatin-vehicle group and *P* = 0.0001 and 0.0002 on days 15 and 25 for cisplatin-ABT263 group). Senescent cells were removed in half the cisplatin-treated mice with 50 mg/kg ABT263 (n = 5, *P* = 0.0271 and 0.0053). Response of the same mice to (**c**) thermal stimulation, as measured by hot plate tests, was measured after cisplatin ± ABT263 treatment (*P* = 0.0027 and 0.1266). The control group (n = 10) received saline and was compared to the cisplatin group without ABT263 treatment (*P* = 0.0004 and 0.0306). (**d**, **e**) Senescent cell removal by ABT263 in the DRG of C57BL/6 mice 72 days after cisplatin treatment as shown by (**d**) p16Ink4a (*P* = 0.0191 and 0.0171) and (**e**) p21 expression (*P* < 0.0001 and < 0.0001). n = 7 or 3. Data points are mean values ± SEM. *P < 0.05, **P < 0.01, ***P < 0.001, and ^****^P < 0.0001 denote significance levels detected by (**b**, **C**) two-way and (**d**, **e**) one-way ANOVA with post-hoc Tukey test.

To verify the role of senescent neuronal cells in CIPN and to test this potential treatment modality using a pharmacologic approach, we next examined whether pharmacological removal of senescent cells by ABT263, a broad-spectrum senolytic agent^42^, reverses CIPN. We induced CIPN in C57BL/6 mice using the treatment described above, and mechanical thresholds were measured on days 15 and 25. Thirty-four days after the second cycle of cisplatin treatment, when CIPN became progressive and irreversible, mice were treated with vehicle or ABT263 as described in the methods section. The longevity of two ABT263 treatment cycles and their efficacy in alleviating CIPN were evaluated over 87 days (Fig. 4a). Mechanical and thermal thresholds were measured on days 65 and 87 after cisplatin treatment to assess the effect of senescent cell clearance on CIPN. The same assays were applied to the control group receiving saline throughout the experiment, while the cisplatin-vehicle group received cisplatin followed by vehicle instead of ABT263. As expected, mice receiving cisplatin developed CIPN, indicated by significantly decreased mechanical thresholds compared to controls (Fig. 4b, *P* = 0.0067 and 0.0103 at day 15 and 25). Interestingly, cisplatin-treated mice recovered basal mechanical thresholds after the first cycle of ABT263 treatment and maintained them following the second ABT263 cycle. Moreover, ABT263-treated mice showed no significant difference in mechanical threshold compared to control mice treated with saline, despite receiving cisplatin (*P* = 0.8644 at day 87). In the absence of ABT263, however, cisplatin-treated mice developed progressive CIPN as shown by steady and significant decreases in mechanical thresholds (Fig. 4b, *P* = 0.0153 and 0.0027 at day 65 and 87). Likewise, cisplatin-treated mice displayed no significant difference in thermal threshold compared to saline-treated controls after receiving two cycles of ABT263 (*P* = 0.9813). In the absence of ABT263, however, cisplatin-treated mice experienced a significant reduction in thermal thresholds (Fig. 4c, *P* = 0.0004 and *P* = 0.0306 after one and two ABT263 cycles). Analysis of mechanical and thermal sensitivity showed no considerable difference between females and males (data not shown).

To provide molecular evidence of the induction of senescent cells in mouse DRG following cisplatin treatment and to verify the effectiveness of ABT263 treatment in senescent cell clearance, p16Ink4a expression was measured in the DRG of C57BL/6 mice. Following cisplatin and ABT263 treatment as outlined in Fig. 4a, DRG were harvested 72 days after the final cisplatin dose and p16Ink4a expression was evaluated via quantification of *Cdkn2a* mRNA. p16Ink4a expression significantly increased with cisplatin if followed by vehicle (Fig. 4d, *P* = 0.0191), but addition of ABT263 after cisplatin returned levels to baseline (*P* = 0.7734), indicating effective pharmacologic clearance of senescent cells. Likewise, a significant cisplatin-induced elevation in p21 expression (Fig. 4e, *P* < 0.0001) was reversed by senolytic treatment with ABT263 (*P* < 0.0001).

Interestingly, GCV or ABT263 in cisplatin-treated mice reversed CIPN as assessed by a restoration of mechanical threshold on day 65 and day 87 (Figs. 3 and 4). Not only were the thresholds equal between the two modalities of senescent cell clearance, but they were equal to the threshold of control mice that never developed CIPN, indicating that pharmacologic and genetic clearance of senescent cells can similarly reverse CIPN, and has the potential to be developed as a novel strategy to mitigate, reverse, and possibly prevent CIPN.

## Discussion

Platinum-based antineoplastic drugs, like cisplatin, commonly cause peripheral neuropathy and significantly impact cancer patients’ quality of life and treatment strategies. We demonstrated that established accumulation of senescent DRG neuronal cells following cisplatin treatment associated with CIPN in C57BL/6 mice. Importantly, senescent cell clearance, both genetically and pharmacologically, reversed CIPN.

While our data show that cisplatin-induced senescence in neuronal cells plays a role in the development of CIPN, other cells, including Schwann cells, macrophages, endothelial cells, and Langerhans cells, could also undergo senescence and contribute to neuropathy. Moreover, the cytokines we measured in DRG can be produced by a variety of senescent cells, not only DRG neurons. Because cisplatin is a systemic treatment, it can affect any organ system, and while distant organs could also impact peripheral neuropathy, we saw no change in senescence-associated serum cytokines (not shown), suggesting this possibility is less likely. Regardless of the involvement of additional cell types, the approach for CIPN therapy by depletion of senescent cells would still be effective in reversing neuropathy.

Cellular senescence induces multiple cellular and molecular changes including development of the SASP. Therapy-induced senescence has been associated with a chronic inflammatory state through increased pro-inflammatory cytokines such as IL-6 and IL-8^11,43,44^. In a recent study of patients with painful peripheral neuropathy, both IL-6 and IL-8 were significantly upregulated in biopsies of the affected skin^45^. Studies have also implicated IL-8 in the development of paclitaxel- and oxaliplatin-induced peripheral neuropathy^46,47^. Similarly, elevated IL-6 expression in the spinal cord and DRG has been shown to play a role in neuropathic pain in animal models^48–50^. The role of IL-6 in chemotherapy-induced peripheral neuropathy, however, is less clear^49^ and showed no relationship to cisplatin treatment in our studies (data not shown). Elucidation of which molecular pathways are involved is worth further investigation.

Assays of known SASP factors in cisplatin-treated DRG neurons revealed an upregulation of *Mmp-*9 mRNA by cisplatin treatment (Fig. 2f). MMPs are implicated in a myriad of biological processes but are most notable for their role in degrading and processing many components of the extracellular matrix^51,52^. They are also widely associated with inflammation and tissue remodeling. Notably, MMP-9 and MMP-2 are thought to be critical in the development of neuropathic pain as they are produced by injured DRG neurons^53^. Interestingly, MMP-9/2 activity significantly increases in DRG following oxaliplatin treatment, and inhibition of these cytokines can alleviate peripheral neuropathy in mice^54,55^. Similarly, MMP-9 accumulated in DRG neurons following paclitaxel treatment, and its inhibition by exogenous TIMP1 or an anti-MMP-9 monoclonal antibody reversed mechanical allodynia in mice^56^. Whether MMP-9 also plays a role in senescence-mediated CIPN is unknown and should be investigated in future studies.

Whether inflammation plays a role in the cisplatin-induced senescent-like changes observed in mice DRG is not yet understood. In fact, the observation that the DRG that acquire senescent-like features do not express ILs commonly associated with the SASP is actually quite interesting. Senescence is typically thought to involve inflammatory factors, however, SASP factors continue being characterized as a cell-type- and senescence-inducer-dependent phenomenon, and there are examples that the SASP is not always associated with inflammatory factors^38^. While MMP-9 was the only measured SASP factor to show a significant increase in gene expression, we do not believe inflammation can be completely excluded as the underlying mechanism due to the fact that an induction in its expression was indeed observed. The elevation of MMP-9 expression is particularly interesting given its presence in injured DRG neurons^53^ and its association with oxaliplatin- and paclitaxel-induced peripheral neuropathy^56,57^. As data accumulates, novel SASP markers in postmitotic cells undergoing non-typical senescent-like changes might be identified. While our results do not conclude that inflammation is the underlying mechanism, they do suggest postmitotic neurons undergo senescent-like changes, albeit not traditional senescence, and removal of these cells reverses CIPN.

Our study examined whether senescent cell clearance can be used as a treatment for CIPN, however, the question remains whether senolytic treatment can be given earlier to prevent CIPN. The rationale for our experiment design was to develop a CIPN therapeutic model to mimic a clinical scenario where only patients who develop CIPN are treated, as only they require reversal of the toxicity. This is because while CIPN is common, not all patients develop the toxicity, and treatment may not need to be given in the absence of symptoms. We anticipate earlier treatment should be effective, once the senescence level is steadily elevated, and would be interested in testing this hypothesis in future studies.

Questions can also be raised about the impact of senescent cell clearance on cancer control. While senescence is characterized by a persistent cell cycle arrest^58^, chronic senescence can paradoxically reduce the tumor response to antineoplastic agents^11^ resulting in increased rates of recurrence and metastasis. Therefore, giving senolytic agents after CIPN has already developed might be less likely to impact tumor control while still being effective against treatment toxicity.

In summary, our data demonstrate that the accumulation of senescent-like neuronal cells in the DRG of mice contributed to CIPN following cisplatin treatment. Elimination of these cells using genetic and pharmacologic approaches reversed the peripheral neuropathy caused by cisplatin. These results provide a basis to investigate the role of senolytic therapy in the prevention and treatment of CIPN in cancer patients needing platinum-based treatment.

## Material and methods

### Mouse strains

C57BL/6 WT mice were purchased from NCI (Charles River Lab, Wilmington, MA). Breeding pairs of *p16-3MR* transgenic mice were kindly provided by Dr. Judith Campisi (Buck Institute for Research on Aging, Novato CA) and bred and maintained in the AAALAC-certified animal facility at the University of Arkansas for Medical Sciences (UAMS). Mice and their progeny received food and water ad libitum and both male and female mice were used at 6–8 weeks of age.

### Mouse dorsal root ganglion (DRG) neuron isolation and culture

Mouse DRG neurons were isolated and cultured as reported by others^59^ with minor modifications. Briefly, the day before DRG dissection, sterilized coverslips in a 12-well cell culture plate were coated with 500 µl poly-L-lysine (PLL, Sigma-Aldrich, catalog number: P1524, 100 µg/ml in water) and incubated at 37 °C overnight. On the day of DRG dissection, a laminin (Thermo Fisher, catalog number: 23017-01) aliquot was slowly thawed on ice and diluted to 2–5 µg/ml in PBS. PLL was then aspirated from each coverslip, and the coverslips were washed 3 times with 500 µl PBS, followed by incubating with diluted laminin at 37 °C until plating DRG neurons.

For DRG isolation, lumbar DRG from 4–5 mice were extracted under a dissecting microscope under sterile conditions, any fibrous material surrounding the DRG was removed and any attached nerve roots cut off. DRG were then transferred to a 15 ml centrifuge tube filled with L15 media (Leibovitz’s L-15, Thermo Fisher, catalog number: 11415-064) followed by centrifugation at 200×*g* at 4 °C for 2 min. DRG were re-suspended in 1.5 ml L15 media containing collagenase (final concentration 1.5 mg/ml, Sigma-Aldrich, catalog number: C-9891) and Dispase II (final concentration 1.5 mg/ml, Roche, catalog number: 04-942-078-001) and incubated at 37 °C for 30 min with gentle, periodic mixing. The enzyme solution was then carefully removed by centrifugation as above, and the pellet re-suspended in 2 ml L15 media and the suspension triturated with a fire-polished glass Pasteur pipette. Next, DNase I was added to the suspension (final concentration 50 µg/ml, Sigma-Aldrich, catalog number: DN25), and the suspension was incubated at 37 °C for 30 min with gentle periodic mixing. Subsequently, trituration was repeated and 5 ml of L15 was added to the suspension, followed by centrifugation at 200×*g* at 4 °C for 5 min and two washings with 5 ml of L15 media each. For DRG purification, 3 ml of 60% Percoll (Sigma-Aldrich, catalog number: P1644) was placed in a 14 ml round bottom tube and carefully overlaid with 3 ml of 30% Percoll. 1 ml of the cell suspension was then carefully placed over the Percoll gradient, followed by centrifugation at 800×*g* for 30 min at 4 °C. DRG neurons resided in the cloudy layer at the interface between the two densities of Percoll after centrifugation. The top 3 ml, which contains myelin and Schwann cells, were gently removed and discarded. The next 2 ml containing DRG neurons were transferred to a 15 ml centrifuge tube and filled with L15 media and centrifuged at 200×*g* for 10 min. DRG were finally re-suspended in 1 ml of Neurobasal-A (Thermo Fisher, catalog number: 10888-022) media supplemented with B-27 (Thermo Fisher, catalog number: 17504-044) and counted using a hemocytometer. For DRG culture, laminin solution from the 12-well cell culture plate with coverslips was aspirated and coverslips were washed 3 times with PBS. After the third wash, 250 µl of cell suspension (containing 10,000 DRG neurons) was immediately plated onto each coverslip and incubated at 37 °C for 1 h before feeding the cells with an additional 250 µl of Neurobasal-A plus B-27.

### Cisplatin treatment of DRG neurons

After overnight culture, 0.5 ml per well of neurobasal-A plus B-27 containing vehicle (0.9% NaCl) or cisplatin was added into the 12-well culture plate that had DRG neurons (cisplatin final concentration 2 µg/ml), and the plate was incubated in a CO_2_ incubator at 37 °C for 5 days.

### SA-β-Gal staining

SA-β-Gal staining was performed using the senescence β–galactosidase staining kit (Cell Signaling Technologies, catalog number 9860) following the manufacturer’s instructions. Briefly, culture media was removed from the cells, which were then rinsed 3 times with 1 ml PBS followed by 1 ml 1× Fixative Solution fixation for 15 min at room temperature, and subsequently twice with PBS. 1 ml of β-Galactosidase Staining Solution was added to each well and incubated at 37 °C overnight in an incubator without CO_2_. The next day, the coverslips were mounted on slides with 70% glycerol and immediately imaged or stored at 4 °C for future imaging using a bright field inverted microscope.

### *p16*^*INK4A*^* and HMGB1 immunofluorescence*

After cisplatin treatment, cell culture media was removed, cells were washed gently three times with PBS, and followed by fixation with cold methanol or 4% paraformaldehyde and blocking with 3% BSA (Sigma-Aldrich, cat#A9647). For p16^INK4A^ immunofluorescence, methanol-fixed cells were incubated with 1:50 Alexafluor594-anti-p16^INK4A^ mouse monoclonal antibody (Santa Cruz, cat#sc-1661 AF594). For HMGB1 immunofluorescence, PFA-fixed cells were incubated with 1:100 anti-HMGB1 rabbit polyclonal antibody (Cell Signaling, cat#3935S), and then with 1:5,000 Alexafluor594-goat anti-rabbit IgG (ThermoFisher, cat#A11037). Coverslips with cells were mounted on clean slides with Prongä-Gold antifade mountant with DAPI (ThermoFisher, cat#P36935). p16^INK4A^ and HMGB1 immunofluorescence were finally analyzed using a Zeiss Fluorescent microscope.

### Quantitative PCR (qPCR)

Total RNA was extracted from lumbar DRG of each mouse using TRIzol™ Reagent (Thermo Fisher, catalog number: 15506026), and reverse transcription was performed with 500 ng total RNA in 20 µl using the High Capacity cDNA Reverse Transcription kit (Thermo Fisher, catalog number: 4368814). Senescence-associated secretory phenotype (SASP) TaqMan™ gene expression assays and internal control GAPDH primers were from Thermo Fisher. For each reaction, 1 μl cDNA was mixed with 10 μl TaqMan™ Fast Advanced Master Mix (Thermo Fisher, catalog number: 4444554) and 1 μl of TaqMan primers. p16INK4A (assay ID: AIBJYQW), p21 (assay ID: Mm00432448_m1), MMP9 (assay ID: Mm00442991_m1), and the internal control mRps2 (Assay ID: MM00475529_m1) were from Thermo Fisher. Samples were added to 8 μl of water (for a total volume of 20 μl) in MicroAmp Fast 96-well Reaction plates (0.1 ml, Thermo Fisher, catalog number: 4349607). All reactions were run in triplicate on an ABI StepOnePlus Real-Time PCR System (Applied Biosystems). SASP gene expression was calculated by the comparative CT method.

### SASP multiplex cytokine/chemokine assay

Three SASP multiplex cytokine/chemokine assay panels, IL-1α/IL-1β/IL-6/CXCL10/TNFα, MMP-3/MMP-13 and TGF-β1 were from Sigma-Aldrich. 75 µl serum was used for each panel as 25 µl triplicates. The assays were run with Bio-plex 200 System from Bio-Rad following the manufacturer’s instructions.

### CIPN mouse model

CIPN was induced by daily intraperitoneal (*i.p*.) injections of cisplatin (50 ml vial, Fresenius KABI, suspended in saline [0.9% sodium chloride, Hospira]) at 2.3 mg/kg for 2 cycles, with 5 consecutive daily injections in each cycle and a 5-day rest between the two cisplatin treatments. On the last injection day of the second cycle and 10 and 50 days thereafter, electronic Von Frey tests were performed to evaluate the mechanical allodynia. Hot plate tests were performed 51 days following the last cisplatin injection to assess thermal sensitivity^33,34,60–62^.

### Tactile allodynia assay (von Frey test)

To assess static mechanical pain hypersensitivity in mice, the thresholds of tactile allodynia were measured with an electronic von Frey system (Dynamic Plantar Aesthesiometer; Ugo Basile, Gemonio, Italy). Mice were placed in a chamber with a mesh screen floor, and a single, unbending filament was applied vertically to the mid-plantar region of both hind paws with increasing force (grams) until a paw-withdrawal response was elicited. The force at which this response occurred was electronically recorded and designated as the paw-withdrawal threshold by the apparatus. These steps were repeated 3 times and the average measurement was calculated and recorded^63,64^.

### Heat hypersensitivity assay (hot plate test)

Heat hypersensitivity was tested using a plantar hot plate analgesia meter, as described^65,66^ (IITC Life Science Inc, CA, USA). Mice were individually placed on a hot plate that was maintained at a temperature of 51.0 ± 0.1 °C. The latency (seconds) to the first sign of hind paw licking or jumping or a jump response to avoid thermal pain was taken as an index of pain threshold and was monitored utilizing an electronic timer. Decreases in withdrawal latency corresponded to increased sensitivity to heat stimuli^67–69^. Results were reported as the mean value of 3 readings.

### Clearance of senescent cells with ABT263 or GCV

C57BL/6 and p16-3MR mice were treated with cisplatin/saline followed by *i.p.* injections of ABT263 or GCV, respectively, to clear cisplatin-induced senescent cells. Following the second cisplatin/saline cycle, mice received 2 cycles of saline, ABT263 (50 mg/kg) or GCV (25 mg/kg) as we reported^42^. Daily *i.p.* injections of the respective treatments were given starting on the 35th day after the last cisplatin injection for 5 consecutive days. After a 16-day rest period, mice received a second 5-day cycle of daily *i.p.* injections. Electronic von Frey tests were performed on the last day of treatment and 10, 50, and 72 days later to evaluate mechanical allodynia. Hot plate tests were performed 51 and 73 days after the last cisplatin/saline injection to assess thermal pain sensitivity.

### Statistics

The behavioral data are presented as a percentage of the naïve mice ± S.E.M of 3–6 mice per group. The results were evaluated using two-tailed Student’s t-test to compare between 2 groups of results and one-way and two-way ANOVA with post-hoc Tukey test to compare among 3 or more groups and to correct for multiple comparisons using GraphPad Prism 6 for Windows. *P < 0.05 was deemed statistically significant.

### Study approval

All procedures were approved by the University of Arkansas for Medical Sciences Institutional Animal Care and Use Committee (IACUC, Little Rock, AR). All animal experiments were performed in accordance with NIH regulations on the use and care of experimental animals.

## Data Availability

All data generated or analyzed during this study are included in this published article.
